# Bryozoans are Major Modern Builders of South Atlantic Oddly Shaped Reefs

**DOI:** 10.1038/s41598-018-27961-6

**Published:** 2018-06-25

**Authors:** Alex C. Bastos, Rodrigo L. Moura, Fernando C. Moraes, Laura S. Vieira, Juan Carlos Braga, Laís V. Ramalho, Gilberto M. Amado-Filho, Ulises R. Magdalena, Jody M. Webster

**Affiliations:** 1 Departamento de Oceanografia, Universidade Federal do Espírito Santo, Avenida Fernando Ferrari 514, Vitória, ES 29090-600 Brazil; 2 Instituto de Biologia and SAGE/COPPE, Universidade Federal do Rio de Janeiro, Ilha do Fundão, Rio de Janeiro, RJ 21944-970 Brazil; 3 Instituto de Pesquisas Jardim Botânico do Rio de Janeiro, Rua Pacheco Leão 915, Rio de Janeiro, RJ 22460-030 Brazil; 4 Programa de Pós Graduação em Dinâmica dos Oceanos e da Terra, Instituto de Geociências, Universidade Federal Fluminense, Avenida General Milton Tavares de Souza, s/n°, Niterói, RJ 24210-346 Brazil; 5 Departamento de Estratigrafía y Paleontología, Universidad de Granada, 18002 Granada, Spain; 6 Geocoastal Research Group, School of Geosciences, The University of Sydney, Sydney, NSW 2006 Australia

## Abstract

In major modern reef regions, either in the Indo-Pacific or the Caribbean, scleractinian corals are described as the main reef framework builders, often associated with crustose coralline algae. We used underwater cores to investigate Late Holocene reef growth and characterise the main framework builders in the Abrolhos Shelf, the largest and richest modern tropical reef complex in the South Western Atlantic, a scientifically underexplored reef province. Rather than a typical coralgal reef, our results show a complex framework building system dominated by bryozoans. Bryozoans were major components in all cores and age intervals (2,000 yrs BP), accounting for up to 44% of the reef framework, while crustose coralline algae and coral accounted for less than 28 and 23%, respectively. Reef accretion rates varied from 2.7 to 0.9 mm yr^−1^, which are similar to typical coralgal reefs. Bryozoan functional groups encompassed 20 taxa and *Celleporaria atlantica* (Busk, 1884) dominated the framework at all cores. While the prevalent mesotrophic conditions may have driven suspension-feeders’ dominance over photoautotrophs and mixotrophs, we propose that a combination of historical factors with the low storm-disturbance regime of the tropical South Atlantic also contributed to the region’s low diversity, and underlies the unique mushroom shape of the Abrolhos pinnacles.

## Introduction

Shallow-water tropical and subtropical reefs are rigid carbonate structures built mainly by corals and crustose coralline algae (CCA) during the Cenozoic^1^. Such living coralgal reefs may reach thousands of square kilometers in shallow tropical and subtropical regions and encompass the world’s most biodiverse marine ecosystems^2^. Reef distribution, structure and morphology vary as a function of the geological, climatic and biogeographic histories of each ocean basin, coupled with modern climate-oceanographic conditions. For instance, benthic community structure, including the relative abundance of reef-builders and eroders, may vary sharply in small spatial scales of a few meters, being influenced by depth, light, nutrient, sedimentation, current and turbulence^3,4^. In addition, biodiversity patterns and community structure also respond to latitudinal and longitudinal gradients of thousands of kilometers, within and among ocean basins^5^.

In the tropical and subtropical portions of the Indo-Pacific and the Caribbean it is well established that reef frameworks are built by species-rich assemblages of scleractinian corals and coralline algae^6,7^. Reef structures develop as well-known morphological types that range from patch and fringing reefs to barrier reefs and atolls^8^. The tropical South-western Atlantic (SWA) represents one of the world’s smallest and poorest coral reef provinces^5,9^, with high endemism levels of up to 50%. In the SWA, the largest and richest coral reefs occur in the Abrolhos Shelf (>8,000 km^2^, 17–19.5°S)^10,11^. The unusual mushroom-shaped morphology of the Abrolhos reefs and the low coral richness of the SWA was noted in the 19^th^ Century^12,13^, but framework building within this entire region remains poorly understood.

**Figure 1:**
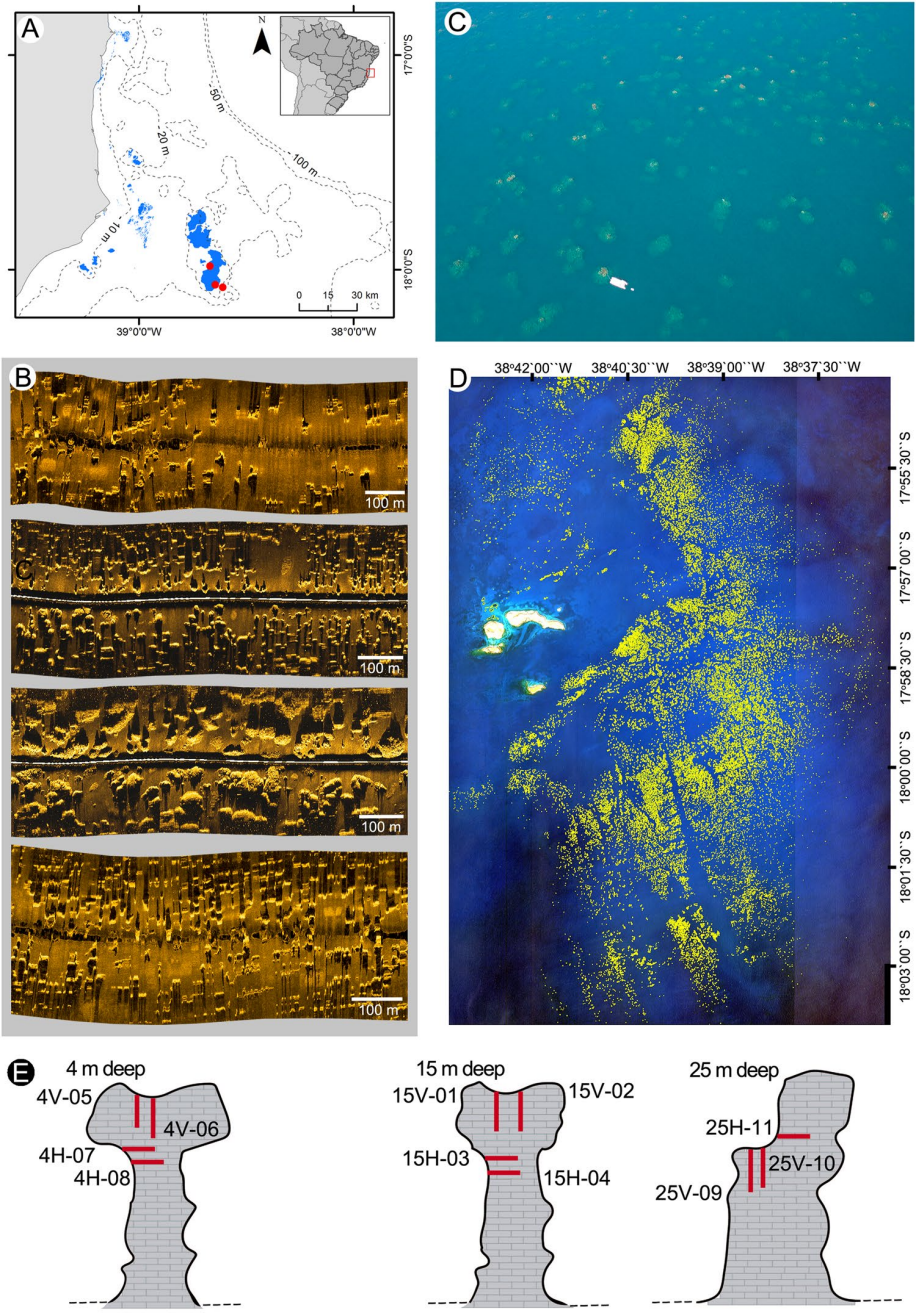
Study area and sampling sites. (**A**) Map showing the Abrolhos shelf physiography and the distribution of reefs in the near-shore and mid-shelf arcs (light blue) and drilling sites (Red dots). (**B**) Side Scan Sonar imagery of mid and outer shelf areas, showing complex submerged reef morphologies. (**C**) Aerial drone image of the “chapeirões” at the Parcel dos Abrolhos (research boat is 20 m long, for scale reference); (**D**) Satelite image (Ikonos 2, 4 m resolution) of Abrolhos Archipelago and its adjacent “chapeirão” reef system (indicated by yellow pixels); (**E**) Schematic view of the 11 cores sampled from the tops and walls of the three reef pinnacles at 4, 15 and 25 m water depth (not on scale). Aerial photo by Fernando Moraes/Rede Abrolhos.

The outer Abrolhos Shelf is largely covered by rhodoliths (CCA-structured nodules) and the inner shelf is covered by siliciclastic-carbonate mixed soft sediments^11,14^. Emergent and quasi-emergent reefs developed during the Holocene and occur in a near-shore and a mid-shelf arc, 12 and 60 km offshore respectively^15^, while mesophotic reefs with erosive topographies occur in the mid-outer shelf and near the shelf edge^11^ (Fig. 1). The major reef builder of the Abrolhos reefs was considered to be the Brazilian-endemic coral *Mussismilia braziliensis* (Verrill, 1868), in association with other corals and CCA^10,16^.

Here we examine the Late Holocene (~2000 yrs BP) framework of reef pinnacles with tops at 4, 15, and 25 m water depth in the Abrolhos Shelf, based on samples obtained from rotary drilling within the mid-shelf arc. We analyzed accretion rates and reef-builder assemblages, and compared our data with available information from Abrolhos near-shore reefs^16^ and elsewhere, within and outside the tropical SWA. We also compiled meteoceanographic historical data in order to support our interpretation of reef morphology, biodiversity and benthic assemblage structure, within the context of the low-disturbance regime of SWA reefs.

## Results

### Pinnacle distribution and morphology

**Figure 2:**
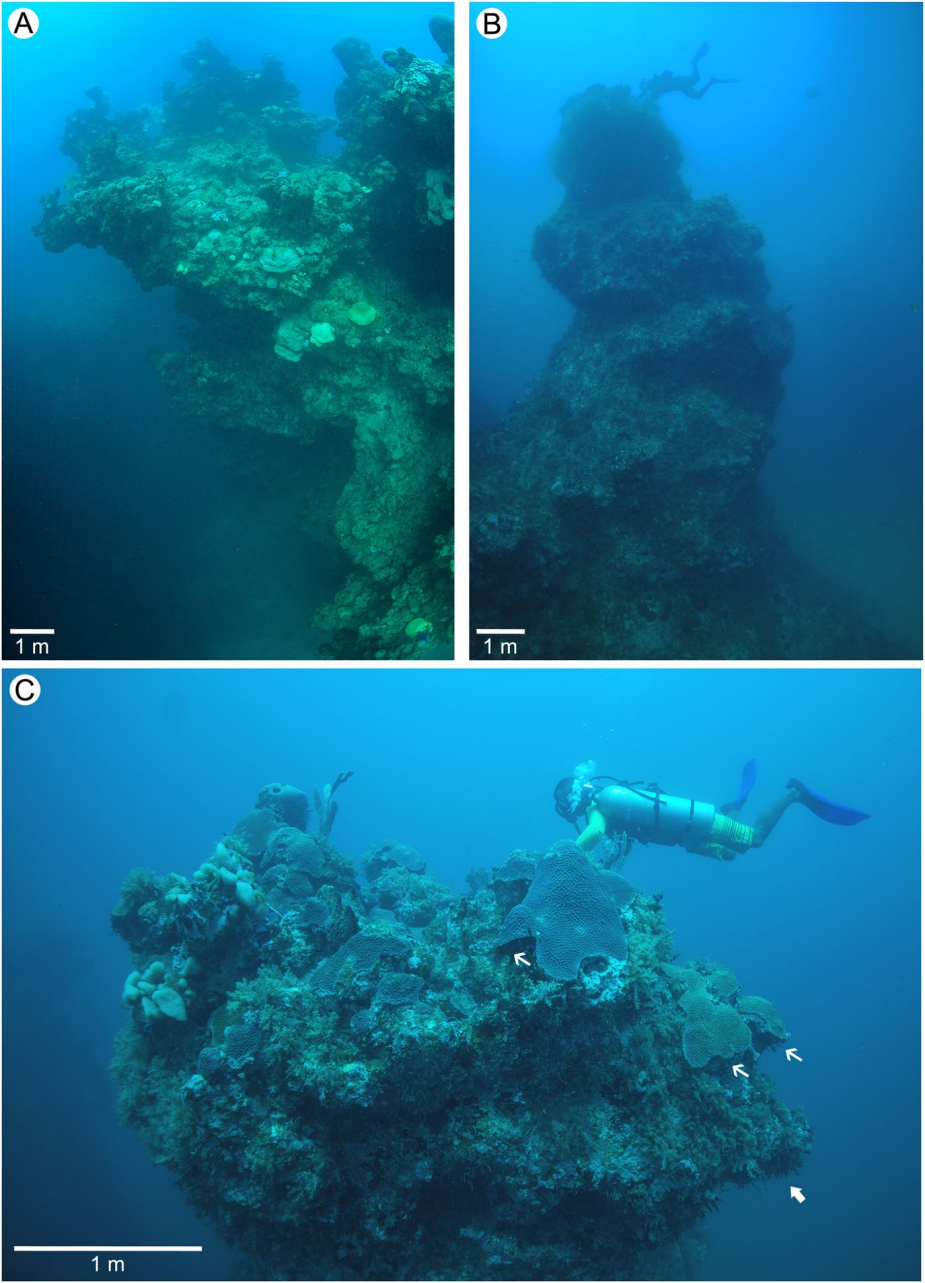
Underwater photos of Abrolhos Bank deep pinnacle reefs in Parcel dos Abrolhos (PA) and California Reef (CR): (**A**) Side view of part of a mushroom shaped “chapeirão” in PA (15 m deep); (**B**) Lateral bottom-up view of a columnar biogenic pinnacle structure in CR (soft bottom at 33 m deep and reef top at 20 m deep); (**C**) Panoramic view of that pinnacle’s reef top in CR, revealing lateral expansion process by the plate coral *Montastraea cavernosa* (arrows). Photos: Áthila Bertoncini & Fernando Moraes/Rede Abrolhos.

Pinnacles were observed along the inner shelf, usually no deeper than 35 m (seabed water depth). These reefs occurred either in a shallow (seabed at 20–25 m, pinnacles tops at 2 to 6 m water depth) and in a deeper margin (seabed at 25–35 m, pinnacles tops at 15 to 25 m water depth) of the reef area. In general, pinnacle distribution showed a greater density in the shallower region, transitioning to more sparsely-distributed pinnacles in the deeper margin of the reef (Fig. 1). Pinnacles in the more central and shallower region are emergent or quasi-emergent and include some structures coalesced in the tops (Fig. 1B–D). These isolated structures consisted of a cylindrical basement from which laterally-expanded tops develop, resembling giant mushrooms (Figs 1E and 2) which are over 20 m high and 40 m across at the tops. Coral richness and cover was highest at the tops, with several important builders restricted to this well-illuminated and relatively flat and shallow habitat (0-10 m depth), including *Mussismilia braziliensis*, *M*. *harttii* (Verrill, 1868) and *Millepora* spp. (Fig. 2 and Supplementary Information Fig. S1). Conversely, pinnacles walls were steep and dim, and a single coral species, *Montastraea cavernosa* (Linnaeus, 1767) was the most conspicuous coral species. The marginal pinnacles with tops below 10 m depth lacked the mushroom-shaped morphology (Figs 1E and 2B) and had smaller coral cover and diversity.

### Reef framework and accretion

**Figure 3:**
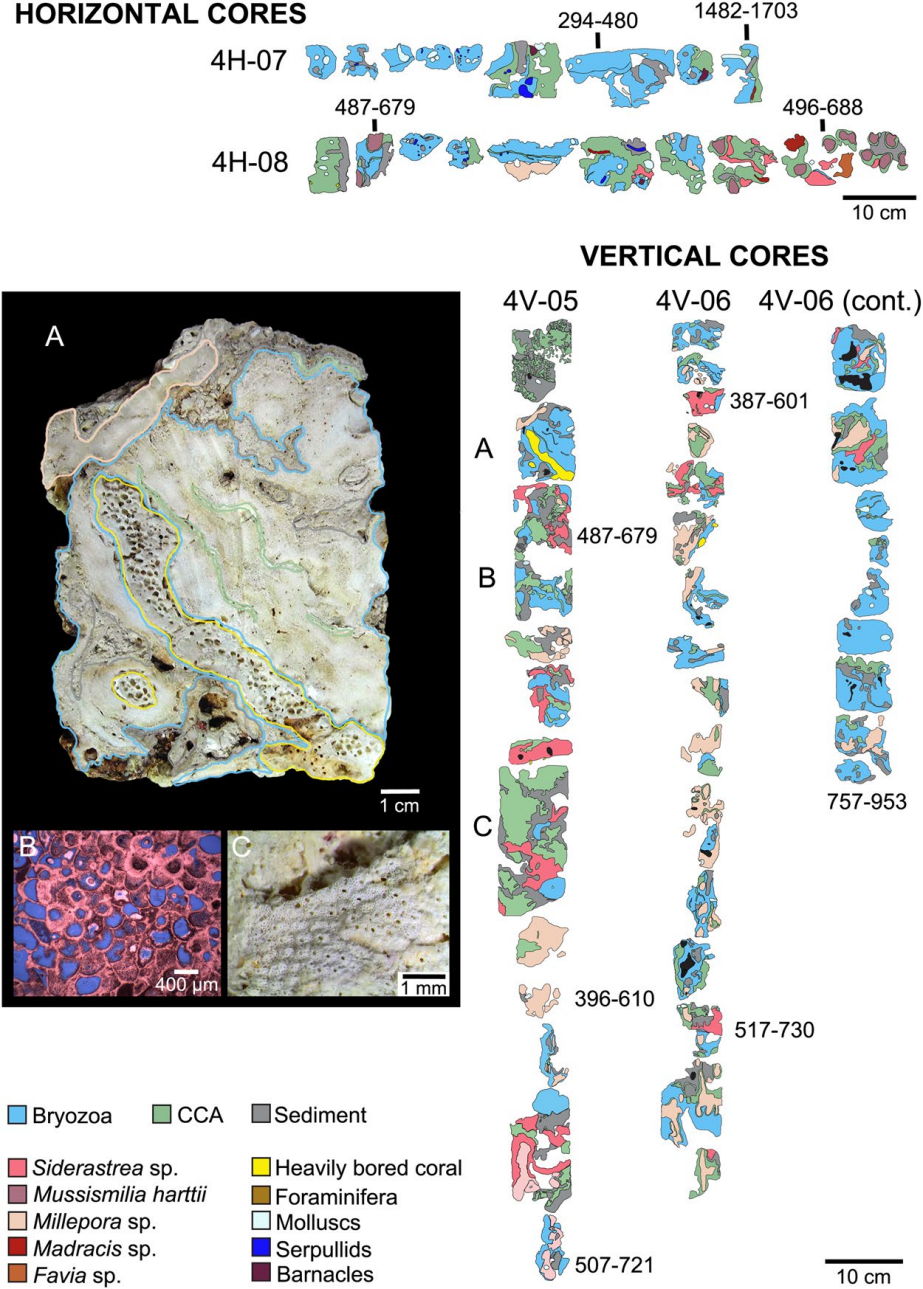
Simplified core log showing the main reef framework types, sedimentary facies, and ^14^C AMS age data collected from Abrolhos reef at 4 m water depth. Vertical (4V-05, 4V-06) and horizontal cores (4H-07, 4H-08) were sampled from the pinnacle’s top and wall, respectively. The stratigraphic distribution of the main reef framework building components was mapped, comprising mainly thickly encrusting cheilostome bryozoans with subordinate amounts of CCAs, corals and sediments (see Methods). The vertical and horizontal accretion rates of the pinnacles were assessed based on calibrated ^14^C AMS measurements from bryozoans and corals in growth position (Methods and Table 1). (**A**) Close-up image of a vertical core sample showing the reef framework dominated by multilayers of encrusting bryozoan colonies; (**B**) Petrographic image showing multilayers of the most abundant bryozoan species forming the reef framework (*Celleporaria atlantica*), including zooids partially filled by cementing matrix; (**C**) Stereomicroscope image of a multilayer colony of *Reptadeonella bipartita*, another important bryozoan species forming the reef framework.

**Figure 4:**
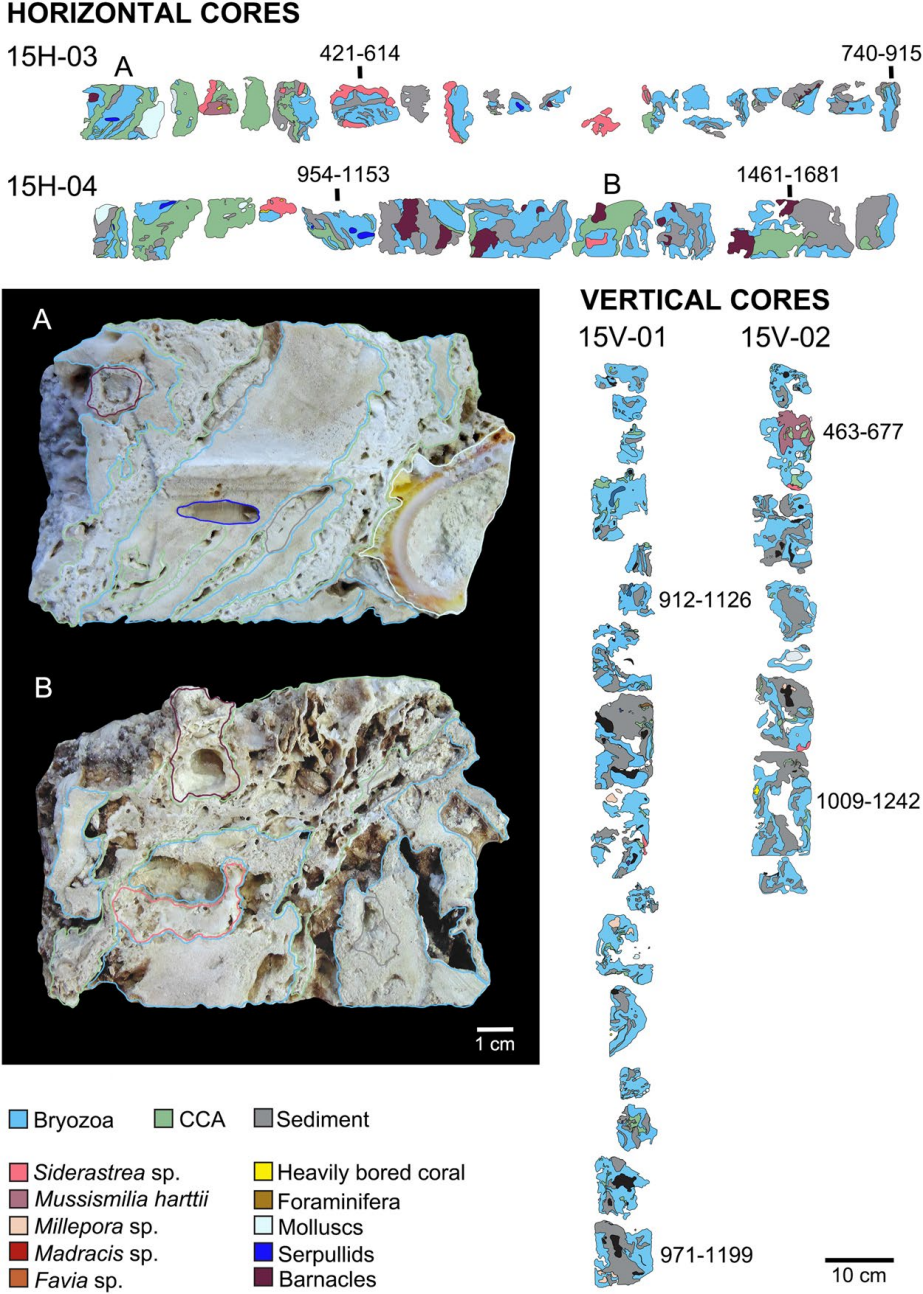
Simplified core log showing the main reef framework types, sedimentary facies, and ^14^C AMS age data collected from Abrolhos reef at 15 m water depth. Vertical (15V-01, 15V-02) and horizontal cores (15H-03, 15H-04) were sampled from the pinnacle’s top and wall, respectively. **(A)** Close-up image of a shallow/younger horizontal core sample showing the reef framework composed mainly by tick multilayers of encrusting bryozoan colonies interspersed with CCA; (**B**) Close-up image of a deeper/older horizontal core sample showing preponderance of bryozoans and CCA over corals in the reef framework building.

**Figure 5:**
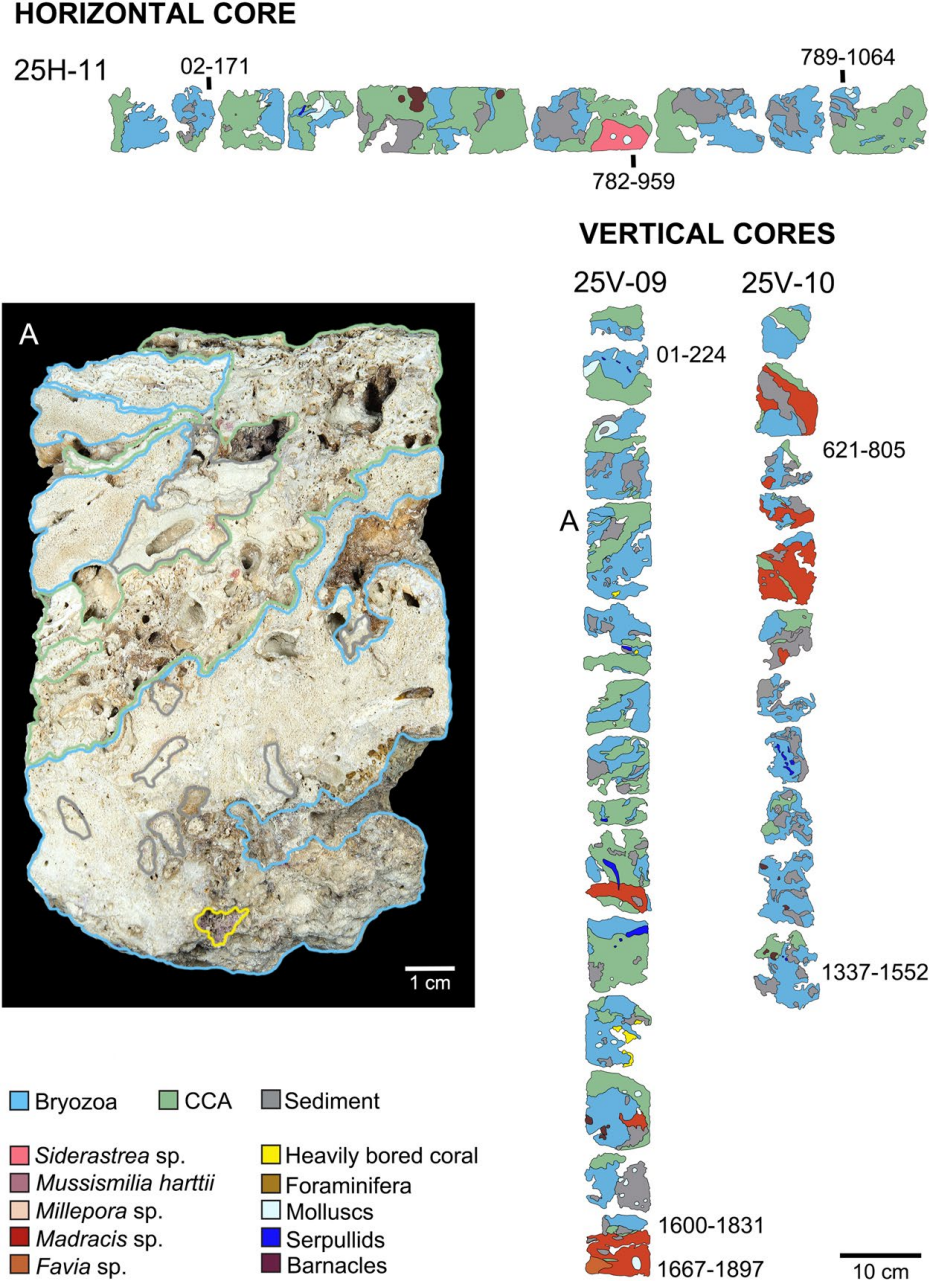
Simplified core log showing the main reef framework types, sedimentary facies, and ^14^C AMS age data collected from Abrolhos reef at 25 m water depth. Vertical (25V-09, 25V-10) and horizontal cores (25H-11) were sampled from the pinnacle’s top and wall, respectively. (**A**) Close-up image of a vertical core sample showing the recurrent pattern of interspersed bryozoans and CCAs in the reef framework. Note sediment fills overgrown by bryozoan layers and remarkable incorporation of fine grains inside the reef matrix.

Based on ^14^C AMS dating of coral, CCA and bryozoan components, reef sequences span 387–757 (4 m depth core), 463–1,199 (15 m depth core), and 224–1,897 cal years BP (25 m depth core) (Figs 3–5). Average vertical accretion rates increased as pinnacle depth decreased, ranging from 0.93 mm.yr^−1^ in the deeper pinnacles (tops at 15 and 25 m depths) to 2.7 mm.yr^−1^ in the shallowest pinnacle (top at 4 m) (Table S1; Supplementary Information Fig. S2 and Table S1). Average horizontal accretion rates varied between 1.4 (4 m), 1.3–2.3 (15 m) and 0.98 mm.yr^−1^ (25 m).

Bryozoans were major components of the reef framework in all cores and age intervals (Figs 3–5; Supplementary Information Table S2). Considering only framework builders, bryozoans comprised 36, 60 and 44% of the reef framework (in 4, 15 and 25 m deep pinnacles, respectively). Bryozoans encompassed at least 20 taxa (11–15 species in each pinnacle), and species composition varied with depth (Table 1). *Celleporaria atlantica* (Busk, 1884) dominated the framework at all cores and depths, followed by two undescribed species, *Parasmittina* sp. nov. 1 and *Stylopoma* sp. nov. 2. Five species occurred at all depths (*C*. *atlantica*, *Hippaliosina imperfecta* (Canu & Bassler, 1928), *Parasmittina* sp. nov. 1, and *Stylopoma* sp. nov. 1 and 2), while three were restricted to the 25 m depth pinnacle (*Steginoporella magnilabris* (Busk, 1854), *Gemelliporina glabra* (Smitt, 1873) and *Metrarabdotos* sp.). Multilayer encrusting was the most common growth form, with 10 species growing as colonies with more than two consecutive layers, six of which with more than 10 layers. Bryozoans were frequently intergrown with CCA. The few erect bryozoan species (*S*. *magnilabris* (Busk, 1854) and *Crisia* sp.) occurred mainly at the 25 m depth pinnacle (Table 1). The former was consistently found inside the cores, while the latter was represented by scattered loose material.

**Table 1:** Bryozoan taxa recorded as main framework reef builders from sampled cores at Abrolhos Bank mid-shelf pinnacles.

Taxons	Drill Depth			Total Presence	Total number of species	Geographic Distribution	References for Distribution	General Shape
	4 m	15 m	25 m					
*Celleporaria atlantica* (Busk, 1884)*	1	1	1	3	5	Brazilian Endemic (RJ, ES, BA)	2; 6	Encrusting
*Parasmittina* sp. nov. 1	1	1	1	3		Abrolhos Endemic	Present Study	Encrusting
*Stylopoma* sp. nov. 1*	1	1	1	3		Abrolhos Endemic	Present Study	Encrusting
*Stylopoma* sp. nov. 2*	1	1	1	3		Abrolhos Endemic	Present Study	Encrusting
*Hippaliosina imperfecta* (Canu & Bassler, 1928)*	1	1	1	3		Brazilian Endemic (RN, BA, ES, RJ)	1; 2; 6	Encrusting
*Parasmittina* sp. nov. 2	1	0	1	2	9	Abrolhos Endemic	Present Study	Encrusting
*Reptadeonella bipartita* (Canu & Bassler, 1928)	1	1	0	2		Caribbean. Brazil (AL, BA, ES)	1; 3	Encrusting
*Rhynchozoon rostratum* (Busk, 1856)*	1	1	0	2		Brazilian Endemic (RN, BA)	1	Encrusting
*Plesiocleidochasma* sp. nov.*	1	1	0	2		Abrolhos Endemic	Present Study	Encrusting
*Utinga castanea* (Busk, 1884)	0	1	1	2		Brazilian Endemic (AL, BA, ES)	1; 4	Encrusting
*Crisia* sp.	0	1	1	2		N/A		Erect
*Labioporella tuberculata* Winston *et al*. 2014*	0	1	1	2		Brazilian Endemic (AL, BA)	2; 5	Encrusting
*Exechonella* sp.	1	1	0	2		N/A		Encrusting
*Metrarabdotos* aff. *jani* Winston *et al*., 2014*	1	0	1	2		N/A		Encrusting
*Hemismittoidea* sp. nov.	0	1	0	1	6	Abrolhos Endemic	Present Study	Encrusting
*Arthropoma cecilii* (Audouin, 1826)	0	1	0	1		Global distribution (South Atlantic, Pacific, Mediterranean, Indian Ocean)	1; 7, 8, 9, 10, 11	Encrusting
*Metrarabdotos* sp.	0	0	1	1		N/A		Erect
*Gemelliporina glabra*	0	0	1	1		Brazilian Endemic (PE, BA, ES)	1; 4	Erect
*Steginoporella magnilabris*	0	0	1	1		Caribbean. Brazil (RJ, ES, BA, AL)	1; 4	Erect
*Crassimarginatella* aff. *tuberosa* (Canu & Bassler, 1928)	0	1	0	1		N/A		Encrusting
**Total: 20 spp**.	**11**	**15**	**13**					

N/A, not applied. Brazilian States Abbreviations: AL, Alagoas; BA, Bahia; ES, Espírito Santo; PE, Pernambuco; RJ, Rio de Janeiro. *Species which colonies formed more than 10 layers inside cores. References for distribution cited in table are in the Supplementary Information: 1, Vieira *et al*., 2008; 2, Winston *et al*., 2014; 3, Almeida *et al*., 2015a; 4, Almeida *et al*., 2015b; 5, Vieira *et al*., 2016; 6, Almeida *et al*., 2017; 7, Canu and Bassler, 1929; 8, Marcus, 1937; 9, Osburn, 1952; 10, Liu, Yin and Ma, 2001.

CCA were the second most abundant framework component (15–28%) and encompassed 12 taxa. The cores from both the shallower and intermediate depth pinnacles’ tops comprised species typical of the shallow-water local assemblage^17^ (e.g. *Porolithon onkodes* (Foslie, 1909), whereas the cores from walls and deeper reefs were dominated by sciaphyllic taxa such as *Sporolithon* (Figs 3–5; Supplementary Information Tables S2 and S3).

Corals and hydrocorals reached 23% of the framework in the shallower pinnacle, decreasing to 9–12% in the deeper ones (Figs 3–5; Supplementary Information Tables S2 and S3). Scleractinians comprised five taxa of encrusting and fast-growing “weedy” corals (e.g. *Favia* sp.), and massive slower-growing “stress-tolerant” species. These latter include both shallow water (e.g. *Mussismilia*) and wider depth-ranging species (e.g. *Madracis*, *Siderastrea*). The only branching forms were fast-growing shallow-water hydrocorals (*Millepora* spp.).

## Discussion

The reef framework of the Abrolhos mid-shelf pinnacles deviates from the archetypal coralgal reefs of the Indo-Pacific and the Caribbean, being dominated by suspension-feeding bryozoans interlayered with CCA, with minor contributions from corals. Instead of being a mere local outlier, this particular framework-building system may be more widespread than previously acknowledged in marginal coralline provinces such as the SWA. For instance, the single coring study from the Abrolhos nearshore reef arc^18^ described a reef framework “dominated by corals”, with bryozoans being “very common and sometimes abundant”. The first study of the drowned reefs off the Amazon river^19^ also described a mixed-framework composed by polychaetes, foraminiferans, bryozoans and mollusks.

Bryozoan contribution on recent reefs generally encompasses low-biomass assemblages of encrusting and cavity-dwelling species that strengthen the structures built by corals and CCA^20^. Encrusting bryozoans are rare on the surface of the Abrolhos pinnacles tops, where CCA and corals dominate the benthic assemblage, being more frequent on the dim walls^21^. Experimental work with colonization plates^22^ revealed that bryozoans were the second most abundant group, following CCA. They were particularly fast-growing on the shaded surfaces of^22^, especially *C*. *atlantica* (Supplementary Information Fig. S1C) and four *Stylopoma* spp. Our cores show that encrusting bryozoans add volume to the framework, going beyond the mere “filling-cavity” role that is widely reported from most modern tropical reefs.

Bryozoan dominance in Late Holocene-modern tropical reefs have rarely been reported. To our knowledge, small patchy reefs built by bryozoans, especially *Celleporaria albirostris* (Smitt, 1873), are only recorded in Joulters Reefs, Bahamas^23^. These reefs thrive in shallow water (4 m deep), with a succession of cheilostome bryozoans (9 species) dominating the base (60–70% of the framework) and corals and CCA in the upper portions.

When bryozoans are considered in the context of longer geological time-scales, the present is not a reliable key to the past^20,24^. Shallow-water reefs dominated by bryozoans were widespread in the Paleozoic^25^, with scleractinian reefs becoming increasingly important since the Late Triassic^26,27^. Concurrently, paleolatitudinal patterns of sediment-producing bryozoans shifted from a pan-tropical distribution in the Paleozoic to a more extratropical range during the Post-Paleozoic^28^. Giant Eocene shelf edge bryozoan mounds are described along the Great Australian Bight, the largest Cenozoic cold-water carbonate province^29,30^. Although temperate and deep assemblages are not a perfect analogue to Abrolhos, increased bryozoan diversity and biomass occurred during Pleistocene lowstands in southern Australia^30^, associated with increased subtropical convergence and upwelling. In Miocene reefs in the Kutai Basin (Indonesia), bryozoans are restricted to coral sheetstone facies and diverse bryozoan assemblage occur encrusting the undersides of thin platy corals embedded in a muddy matrix^31,32^. These species-rich coral communities formed low-relief buildups lacking rigid frameworks in nearshore turbid environments^31,33^. By contrast, bryozoans are absent in deposits with high siliciclastic input, and in rigid frameworks of tabulate and branching corals and coralline algae, which formed under lower terrestrial influx^31,32^.

Modern bryozoans occur at all latitudes, but tend to form significant accumulations only in eutrophic/turbid and deep/cold waters^20,34,35^. The Abrolhos reefs are within 15–30 m water depths and under 21.7–29.6 °C, i.e. within the ranges where coralgal reef growth predominates circutropically^36^. Bryozoan dominance in the reef framework seems to be primarily associated to the region high nutrient and turbidity. The eastern coast of tropical South America receives substantial riverine input and terrigenous sedimentation^10,37^. Dissolved inorganic nitrogen and phosphorous concentrations in Abrolhos are above commonly assumed eutrophication thresholds^38^, and phytoplankton productivity is significantly high^39^. Remote sensing-derived chlorophyll *a* annual values range from 0.38 to 0.79 mg.m^−3^ ^40^, corresponding to a persistent mesotrophic condition^41^ that is enhanced by shelf-edge upwelling during the wet season (spring/summer). Turbidity is overall high and increases seasonally due to re-suspension of biogenic sediments^42^. Therefore, oceanographic conditions deviate sharply from those of the archetypal Indo-Pacific and Caribbean coralgal reefs, determining the unique reef-building assemblages. With the exception of oceanic islands^43,44^, Brazilian reefs develop under similar high turbidity and nutrient levels^15,19^, comprising foremost examples of turbid zone reefs with low diversity but significant coral cover^45,46^. Nevertheless, modern and ancient examples include turbid zone reefs that can present high coral diversity^3,46^, and even high bryozoan diversity^31,32^, but the latter largely occur at low biomasses and encrusting underneath coral surfaces^31^, which deviates from the high bryozoan abundance recorded in the Abrolhos mid-shelf reefs.

**Figure 6:**
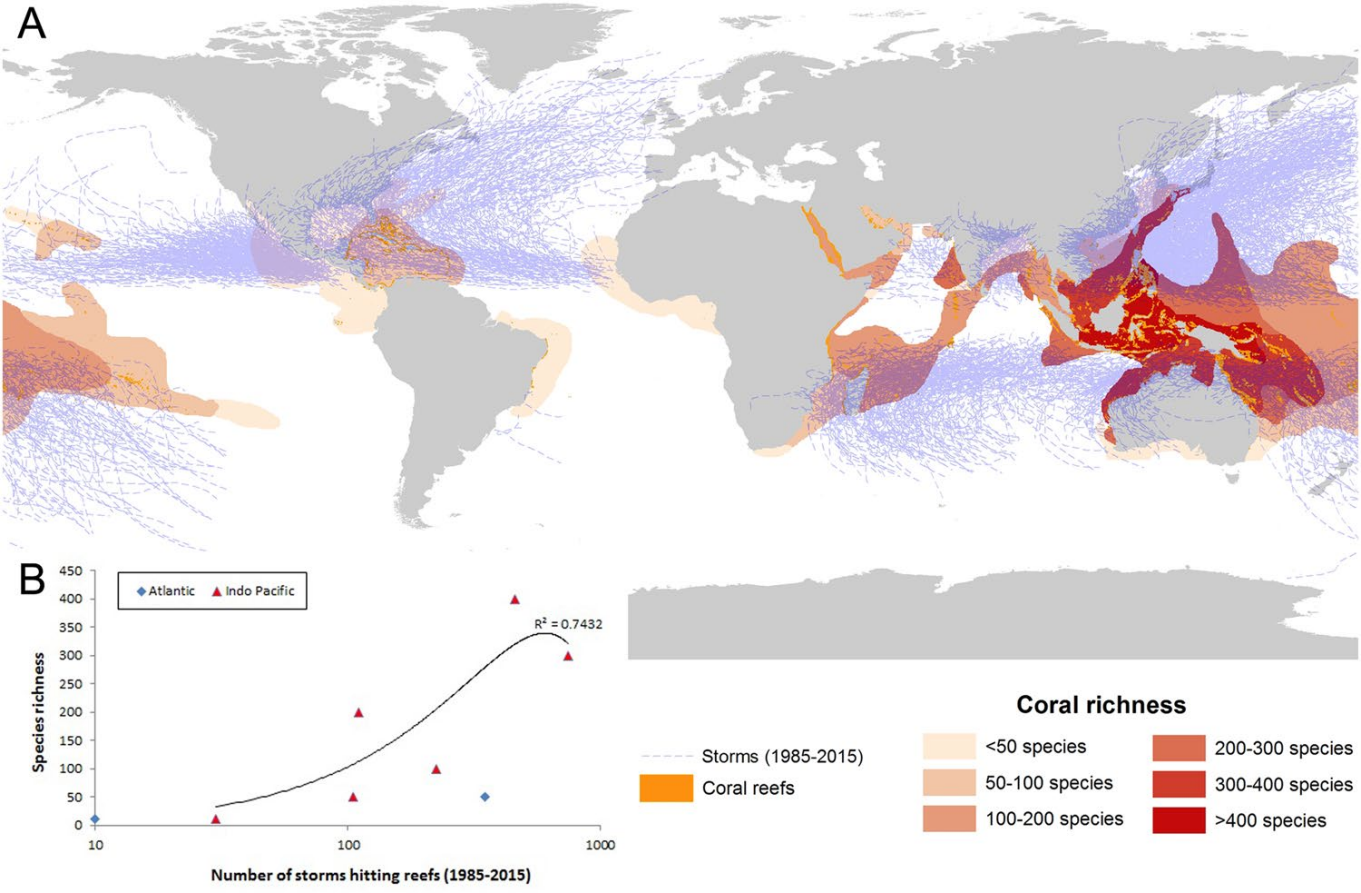
(**A**) World occurrence of storm track records from 1985–2015 superposed on coral richness of all reef provinces, (**B**) Graph showing the relationship between coral species richness and number of tropical storms/hurricanes.

Another potential driver of reef growth and diversity along the eastern Brazilian margin is the region’s low disturbance regime, with very rare tropical storms and hurricanes (Fig. 6). Akin to tropical rainforests, disturbance from extreme wave and wind energy events may play an important role in coral reef diversity^47,48^, creating large habitat patches under different succession stages. Such extreme events change reef morphology and also preclude dominance by slow-growing “stress-tolerant” species. While historical factors (e.g. isolation, glacial compression) may underlie the low species richness and functional diversity of SWA coral assemblages, the marginal conditions (e.g. turbidity) and low disturbance regime may be major drivers of the region’s low coral diversity. In addition, the low disturbance regime does not impose significant constraints (e.g., physical erosion) to horizontal accretion of carbonate framework, preventing the breakage of the huge lateral expansions of the oddly shaped pinnacles. Previous mechanisms explaining the mushroom-shaped morphology of the Abrolhos reefs accounted only for the limitations in upward accretion due to the sea level drop during the last 4,000 years^10^. However, while sea level plays a major role in reef growth, it does not seem to be the single driver of the peculiar morphology of the Abrolhos reefs. Flat pinnacles tops occur in the subsurface, at depths of up to 10 m, and our results indicate ongoing vertical growth. For instance, a depth vs age analysis from the shallower pinnacle (4 m deep x 910–1310 Cal yrs BP) does not support a sea-level control of its shape^10^.

Despite being relatively small and species-poor, tropical SWA reefs provide relevant insights on global patterns of reef building and on the meso-scale drivers of reef biodiversity. Our results challenge the coralgal-framework paradigm of modern shallow water tropical reef building. The Abrolhos mid-shelf reefs deviate from coral reefs in which mixo- and autotrophic organisms (scleractinian corals and CCA) are the main builders. Instead, at least during the last 2,000 yr., these reefs have been largely built by bryozoans, which are heterotrophic suspension-feeders, with a secondary role of CCA and a species-poor coral assemblage. These findings suggest that the role played by bryozoans in reef building may be underestimated in other shallow water tropical reef systems under mesotrophic conditions.

## Methods

### Study region

Detailed descriptions of the Abrolhos Shelf reefs (Fig. 1) are provided by^11,14^, including the side scan images in Fig. 1B. Besides using literature data to describe reef morphology and benthic assemblage structure, we carried out a photographic assessment, both underwater (by SCUBA diving) and aerial (by drone) (Fig. 1D). Reefs in the mid-shelf arc are locally called “chapeirões” (=large hats), accounting for their unique “mushroom-like” morphology that was remarked since the first scientific surveys^12,13^. The mid-shelf chapeirões consist of 2–20 m height pinnacles, with diameters of 2–50 m and laterally expanded tops near the surface. Near-shore, pinnacles are shorter but similarly shaped, often coalescing to form extensive shallow banks and patch reefs^15^.

### Field sampling and sample processing

Coring was carried out using a diver-operated, submarine hydraulic rotary drill. Cores were taken in three depths (4, 15 and 25 m) and habitats (pinnacles’ tops and walls) along the mid-shelf arc. For each depth and habitat, two along side cores were taken, with the exception of the 25 m deep pinnacle wall, where only one core was recovered. All cores were sliced in two halves and photographed in high resolution. The main reef framework types, sedimentary facies and sample context in the cores were logged using a combination of sample material, petrographic thin sections and digital images following procedures laid out in^49^. Core recoveries and depths were calculated by moving the recovered samples to the top of each core run, following standard International Ocean Discovery Program (IODP) protocols^50^. The high resolution core images were displayed in Corelyzer 2.0.2^50^ and the percent surface area of each of the main lithologies and reef framework types calculated.

Drilled subsamples of specific lithologies (corals, bryozoans or coralline algae) were radiocarbon dated at Beta Analytics-Florida (USA) via Accelerator Mass Spectrometry (AMS). Dates are reported as calendar years BP (“present” = 1950 CE) using the 2 sigma confidence interval. Calibration was carried out using Calib 7.1 available at http://calib.qub.ac.uk/calib. The Marine13 calibration curve was applied assuming a global marine reservoir effect of 400 years and a Delta R of 85 ± 25 (average value for the two closest localities) for regional correction. Only samples interpreted as *in situ* (i.e. original growth position) were chosen for dating purposes. Vertical accretion rates were calculated using mean ages and the stacked core sample depths. The maximum potential depth error of each dated core sample was directly related to the actual core recovery of each core run (recovery error in Supplementary Table 1). Only dated samples that were outside of their respective ages errors were used to calculate accretion rates.

All the processed data, including drilling sites coordinates, C14 results, core composition and taxa information are provided as tables in the Supplementary Information file.

### Secondary datasets and statistical analyzes

The storm dataset was compiled from NOAA National Climatic Data Center, World Data Centre for Meteorology - https://www.ncdc.noaa.gov/ibtracs/index.php?name=wmo-data, spanning from 1985 and 2015. Global level scleractinian coral richness data was compiled from^3^ and digitalized as shape files representing layers with <50, 51–100, 101–200, 201–300 and >400 species. Satellite image is an Ikonos - 2, with spatial resolution of 4 m. A supervised classification was carried out using Mahalanobis Distance algorithm^51^. All explicit spatially data were introduced and processed in a Geographic Information System (GIS).

### Data Availability

All data generated or analysed during this study are included in this published article and its Supplementary Information files.

## Electronic supplementary material

supplementary information
