# Supplementary material for: Bryozoans are Major Modern Builders of South Atlantic Oddly Shaped Reefs
Source: Sci Rep. 2018 Jun 25;8:9638. doi: 10.1038/s41598-018-27961-6 (PMC6018419; doi:10.1038/s41598-018-27961-6)
Supplement: Supplementary file 1 — supplementary information [file 41598_2018_27961_MOESM1_ESM.doc]

**Supplementary Information for**

**Bryozoans are Major Modern Builders of South Atlantic Oddly Shaped Reefs**

*Alex C. Bastos^a^, Rodrigo L. Moura^b^, Fernando C. Moraes^c^, Laura S. Vieira^d^, Juan Carlos Braga^e^, Laís V. Ramalho^c^, Gilberto M. Amado-Filho^c^, Ulises R. Magdalena^b^, Jody M. Webster^f^

Supplementary Figures 1-2

Supplementary Tables 1-3

Supplementary Information – References Table 1

**Supplementary Figures**

**
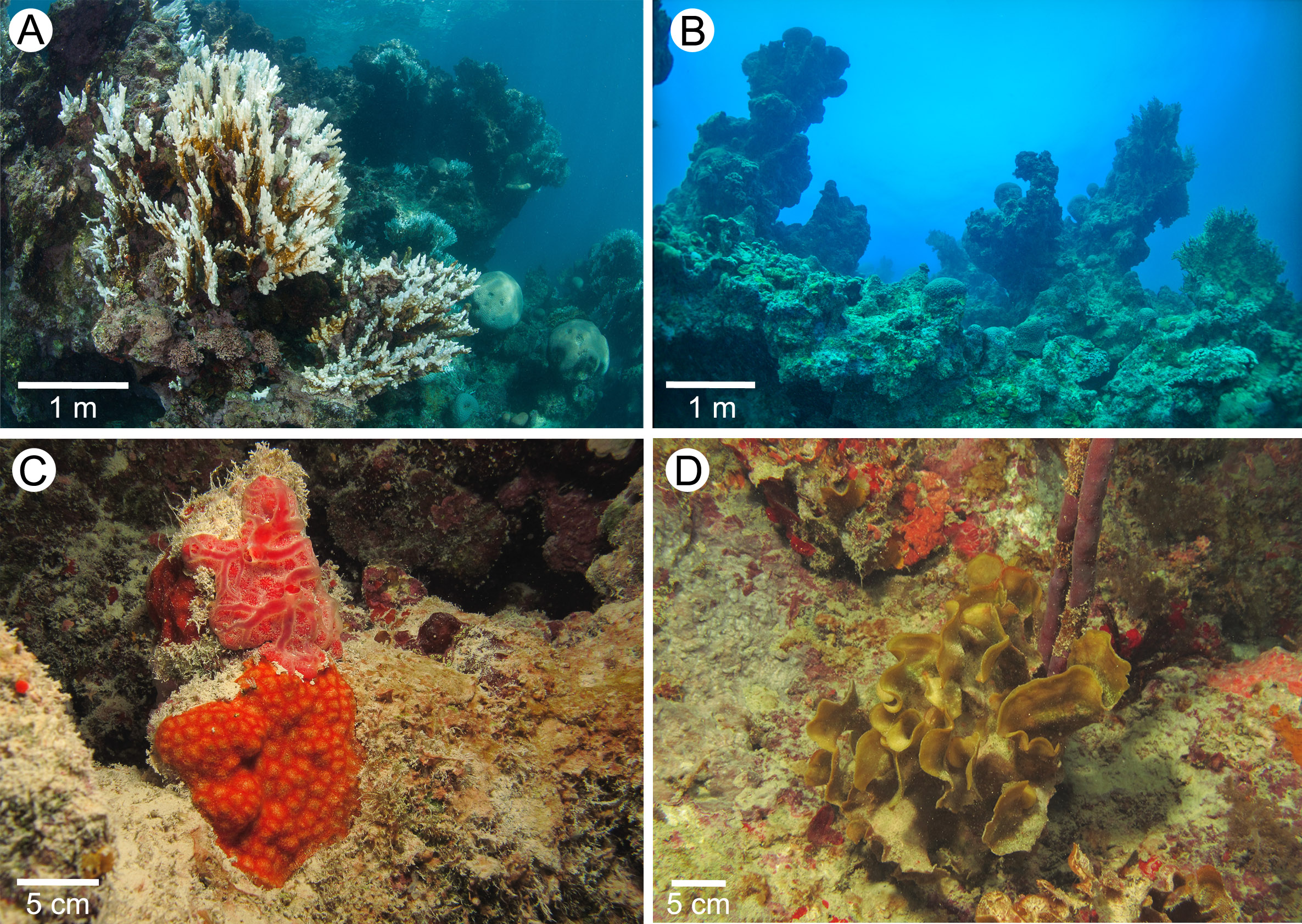
**

**Supplementary Figure S1: Panoramic underwater photographies of reef habitats and close-up of bryozoan key species: (A) Shallow reef top at 4 m water depth, showing *Millepora* spp. (fire coral, bleached in 2016), in the shallowest plane and *Mussismilia braziliensis* brain-coral colonies on the deeper part; (B) Biogenic columnar formations on a “chapeirão” edge at 15 m water depth; (C) The most abundant Bryozoa species *Celleporaria atlantica* growing as an encruster on a vertical wall (15 m deep), with the sponge *Monanchora arbuscular* just above it; (D) Arborescent Bryozoa *Steginoporella magnilabris* on a horizontal ledge (30 m deep). Photos by Áthila Bertoncini & Fernando Moraes/ Rede Abrolhos.**

**Supplementary Figure S2. Combined ^14^C AMS age vs depth data from the reef cores collected vertically from the Abrolhos reef at 4 m, 15 m and 25 m water depth.** See Methods in the main text for a description of how the samples were selected, dated and calibrated and then how the vertical and horizontal reef accretion rates were calculated (Supplementary Table 1). Note that the linear trend line was fitted to only those data that were outside of their respective age errors.

**Supplementary Tables**

**Supplementary Table 1. ^14^C AMS age data from reef cores collected from the Abrolhos reef at 4 m, 15 m and 25 m water depth.** See Methods for the detail of how the vertical and horizontal reef accretion rates were calculated (Datum WGS 84).

**Supplementary Table S2**. Percentage values of the main framework components (lithology) of the Abrolhos reefs sampled cores. Note: “other taxa” category aggregates Barnacle, Foraminifera, Heavily bored corals, Molluscs, and Serpullids.

**Supplementary Table S3. Coral and coralline algae species comprising the Abrolhos reef cores.**

**Supplementary Information – Reference List Table 1**

1 Vieira et al., 2008;

Vieira, L.M., et al. Synopsis and annotated checklist of recent marine Bryozoa from Brazil. Zootaxa, 1810, 1–39, (2008).

2 Winston et al., 2014;

Winston, J.E. et al. Scientific results of the Hassler Expedition. Bryozoa. No. 2. Brazil. Bulletin of the Museum of Comparative Zoology, 161, 139–239, (2014).

3 Almeida et al., 2015a;

Almeida, A.C.S. et al. Taxonomy of recent Adeonidae (Bryozoa, Cheilostomata) from Brazil, with the description of four new species. Zootaxa, 4013 (3), 348–368, (2015).

4 Almeida et al., 2015b;

Almeida, A.C. et al. Gymnolaemata bryozoans of Bahia State, Brazil. Journal of the Marine Biological Association of the United Kingdom 8: e120. doi:10.1017/S1755267215000743 (2015).

5 Vieira et al., 2016;

Vieira, L.M., et al. Taxonomy of intertidal cheilostome Bryozoa of Maceió, northeastern Brazil. Part 1: Suborders Inovicellina, Malacostegina and Thalamoporellina. Zootaxa, 4097, 59–83, (2016).

6 Almeida et al., 2017.

Almeida, A.C.S., et al. Diversity of marine bryozoans inhabiting demosponges in northeastern Brazil. Zootaxa, 4290, 281–323, (2017).

7 Canu and Bassler, 1929;

Canu, F. and Basslek, R.S. Les Bryozotiires du Maroc et de. Mauritanie, Memoires de la Society des Sciences naturelles du Maroc, 10, p. 23. (Biology.), (1929).

8, Marcus, 1937;

Marcus, Bryozoarios Marinhos Brasileiros I. Boletim Faculdade. Philosophia Cièncias e Letras, Universidad de São Paulo, 1, Zool. 1, p. 1-224, (1937).

9, Osburn, 1952

Osburn, R.C. Bryozoa of the Pacific coast of America, part 2, Cheilostomata-Ascophora.

Allan Hancock Pacific. Expeditions, 14, 271–611, (1952).

11, Liu, Yin and Ma, 2001

Liu, X. et al. Biology of Marine-Fouling Bryozoans in the Coastal Waters of China

. Science Press, Beijing, 860 pp (2001).
